# Nomogram for the preoperative prediction of Ki-67 expression and prognosis in stage IA lung adenocarcinoma based on clinical and multi-slice spiral computed tomography features

**DOI:** 10.1186/s12880-024-01305-5

**Published:** 2024-06-12

**Authors:** Zhengteng Li, Hongmei Liu, Min Wang, Xiankai Wang, Dongmei Pan, Aidong Ma, Yang Chen

**Affiliations:** 1 Department of Radiology, Jining No.1 People’s Hospital, No. 6 Jiankang Road, Rencheng District, Jining, 272000 China; 2 Thyroid and Breast Surgery, Jining No.1 People’s Hospital, No. 6 Jiankang Road, Rencheng District, Jining, 272000 China; 3 Department of Radiology, Yantai Yeda Hospital, Yantai Economic and Technological Development Zone, No. 11 Taishan Road, Yantai, 264000 China

**Keywords:** Lung adenocarcinoma, Ki-67, Tomography, X-ray computed tomography, Nomogram

## Abstract

**Objective:**

This study developed and validated a nomogram utilizing clinical and multi-slice spiral computed tomography (MSCT) features for the preoperative prediction of Ki-67 expression in stage IA lung adenocarcinoma. Additionally, we assessed the predictive accuracy of Ki-67 expression levels, as determined by our model, in estimating the prognosis of stage IA lung adenocarcinoma.

**Materials and methods:**

We retrospectively analyzed data from 395 patients with pathologically confirmed stage IA lung adenocarcinoma. A total of 322 patients were divided into training and internal validation groups at a 6:4 ratio, whereas the remaining 73 patients composed the external validation group. According to the pathological results, the patients were classified into high and low Ki-67 labeling index (LI) groups. Clinical and CT features were subjected to statistical analysis. The training group was used to construct a predictive model through logistic regression and to formulate a nomogram. The nomogram’s predictive ability and goodness-of-fit were assessed. Internal and external validations were performed, and clinical utility was evaluated. Finally, the recurrence-free survival (RFS) rates were compared.

**Results:**

In the training group, sex, age, tumor density type, tumor-lung interface, lobulation, spiculation, pleural indentation, and maximum nodule diameter differed significantly between patients with high and low Ki-67 LI. Multivariate logistic regression analysis revealed that sex, tumor density, and maximum nodule diameter were significantly associated with high Ki-67 expression in stage IA lung adenocarcinoma. The calibration curves closely resembled the standard curves, indicating the excellent discrimination and accuracy of the model. Decision curve analysis revealed favorable clinical utility. Patients with a nomogram-predicted high Ki-67 LI exhibited worse RFS.

**Conclusion:**

The nomogram utilizing clinical and CT features for the preoperative prediction of Ki-67 expression in stage IA lung adenocarcinoma demonstrated excellent performance, clinical utility, and prognostic significance, suggesting that this nomogram is a noninvasive personalized approach for the preoperative prediction of Ki-67 expression.

## Introduction

Lung cancer is the leading cause of cancer-related deaths globally. Despite the widespread use of low-dose computed tomography (CT) for lung cancer screening in recent years, the 5-year survival rate for patients with early-stage lung cancer remains at only 59% [1]. Non-small cell lung cancer (NSCLC) accounts for > 85% of all lung cancer cases, with lung adenocarcinoma (LUAD) being the most common histological subtype. Surgical resection is the most effective treatment for stage IA NSCLC; however, the choice of specific surgical procedure is debated [2].

The Ki-67 nuclear protein is a key marker used to assess cell proliferation and tumor heterogeneity. Its expression levels rapidly increase from the G1 phase to the mitotic phase and are crucial indicators for assessing tumor malignancy and patient disease-free survival, recurrence-free survival, and overall survival [3–6]. Ki-67 expression is widely used in the diagnosis and treatment of malignant tumors to select surgical approaches, design targeted therapies, and evaluate prognosis [7]. A recent study demonstrated that rapid Ki-67 immunohistochemistry during surgery can be used to identify stage IA NSCLC patients with a greater risk of recurrence to aid in the selection of appropriate surgical strategies [8]. Additionally, this study suggested that segmentectomy may be a more reasonable choice than lobectomy for early-stage, low-grade NSCLC.

However, traditional Ki-67 testing requires invasive methods such as biopsy or surgery to obtain specimens. Moreover, due to tumor heterogeneity, biopsies may lead to sampling errors, resulting in an underestimation of the disease. Furthermore, this method may increase the incidence of pneumothorax by approximately 15% [9]. High expression of Ki-67 is an unfavorable factor affecting progression-free survival (PFS) [10]. Therefore, developing a noninvasive method to accurately predict Ki-67 expression in stage IA LUAD preoperatively is highly important.

Currently, multi-slice spiral computed tomography (MSCT) is the primary method for diagnosing and monitoring lung cancer. Some MSCT features of LUAD are closely related to Ki-67 and can be used to predict tumor differentiation and the Ki-67 expression level [11–13]. In addition, the Ki-67 proliferation index (PI) is an independent risk factor for recurrence in patients with early-stage LUAD after segmentectomy [14]. However, no study has investigated the suitability of a nomogram based on CT features for predicting the Ki-67 status of stage IA LUAD [15–17], although previous studies have demonstrated the association of the Ki-67 expression level with the malignant potential and prognosis of stage IA LUAD, and relevant studies have proven that thin-section CT findings of peripheral lung adenocarcinomas correlate well with histologic prognostic factors [18]. Nomograms visualize regression equations by integrating multiple predictive indicators through multiple-factor regression analysis, providing personalized and highly accurate risk assessments [19].

In the present study, we developed and validated a nomogram based on clinical and MSCT features for the preoperative prediction of Ki-67 expression in stage IA LUAD. The nomogram was internally and externally validated and is intended to create a more practical and widely applicable predictive model to assist in the clinical diagnosis and treatment of stage IA LUAD.

## Materials and methods

### Patient characteristics

Data were collected retrospectively from patients diagnosed with stage IA LUAD who underwent surgical procedures between January 2016 and February 2023 at Jining No.1 People’s Hospital and Yantai Yeda Hospital. The data included clinical, pathological, and radiological information. The inclusion criteria were patients (i) who underwent complete surgical resection with postoperative pathological confirmation of LUAD and complete medical records; (ii) who underwent thin-slice CT scanning with an imaging-to-surgery time interval not exceeding 2 weeks; and (iii) who had not received any prior diagnostic or therapeutic interventions such as biopsies, radiation therapy, or chemotherapy before surgery. The exclusion criteria were patients who (I) had concurrent or previous malignancies at other anatomical sites (7 patients); (II) had multiple synchronous LUADs (15 patients); or (III) for whom CT imaging could not clearly visualize the lesion or showed suboptimal image quality that prevented analysis (8 patients).

**Fig. 1:**
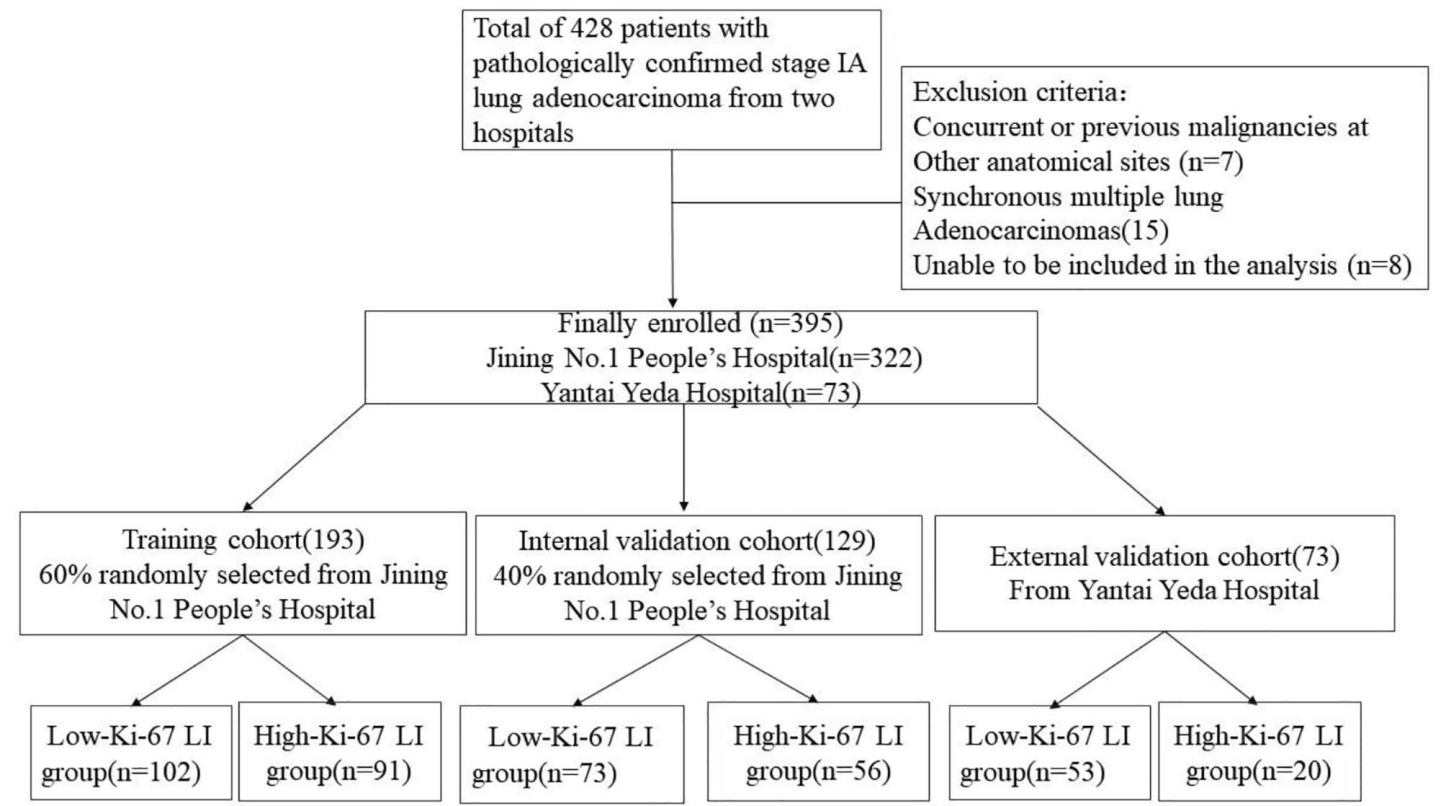
Study flow diagram

The inclusion and exclusion criteria were applied at the two centers, and 395 patients out of the 428 patients were ultimately enrolled. The 322 patients treated at Jining No.1 Patients at People’s Hospital between January 2018 and February 2023 were divided into training and internal validation cohorts at a 6:4 ratio. Additionally, 73 patients treated at Yantai Yeda Hospital between May 2019 and February 2022 were assigned to the external validation cohort (Fig. 1).

This study was approved by the Ethics Committees of Jining No.1 People’s Hospital and Yantai Yeda Hospital.

### CT scans

Chest CT images were obtained using multirow spiral CT scanners (Somatom Definition Flash CT or Somatom Definition AS CT; Siemens Healthineers, Erlangen, Germany) using the following scan parameters: 100–120 Kv tube voltage, automatic tube current modulation, 512 × 512 matrix, 500 mm field of view, and 1–2 mm slice thickness. All CT data were obtained with full inspiration in the supine position. Digital Imaging and Communications in Medicine images were transferred to a picture archiving and communication system.

### Clinical data and CT scan analysis

The general clinical data typically included sex, age, symptoms, and smoking history.

Two radiologists (Li and Chen, with 12 and 10 years of experience in chest radiology, respectively) independently evaluated the following CT features: lobe location, tumor density type (solid, mixed ground-glass opacities [mGGO], and pure GGO [pGGO]), shape, tumor-lung interface (distinct, indistinct), lobulation, spiculation, vacuole, pleural indentation, bronchovascular bundle sign, air bronchogram, and maximum nodule diameter (largest section on the axial position). Discrepancies between the two radiologists were resolved by consulting a third radiologist (Wang, with 15 years of experience in chest radiology). Although all three radiologists knew that the patients had stage IA LUAD, they were blinded to the pathological results. To determine the interobserver reproducibility of CT feature extraction, Cohen’s kappa coefficient and the intraclass correlation coefficient (ICC) were calculated. Features with poor consistency (Cohen’s kappa or ICC < 0.8) were excluded.

### Pathology

All specimens were surgically resected to ensure that Ki-67 PI represented the whole tumor, and a mouse anti-human Ki-67 monoclonal antibody (Zhongbin Jinqiao Biotechnology Co., Ltd., Beijing, China) was used for immunohistochemical staining in strict accordance with the manufacturer’s instructions. Two to three tumor sections with the hottest spots were selected, and at least five high-power fields were randomly selected from each stained section. The average percentage of positive cells among the total number of cells was quantitatively analyzed using the Ki-67 PI. The patients were categorized according to the Ki-67 labeling index (LI), with high and low Ki-67 LI defined as Ki-67 PI ≥ 15% and < 15%, respectively [20]. All pathological diagnoses were independently made by two pathologists. In cases of disagreement, a consensus was reached through discussion.

### Follow-up

We conducted long-term follow-up telephone calls and medical records until November 1, 2023, for patients who underwent radical surgery. Patients underwent chest CT scans every 6 months after surgery and then annually if no tumor progression was observed for 2 years. Recurrence-free survival (RFS) was defined as the time from surgery to recurrence.

### Statistical analysis

IBM SPSS Statistics for Windows, version 26.0, and R software, version 3.6.3 (http://www.R-project.org), were used to process the data. The Kolmogorov–Smirnov test was used to test the normality of continuous variables. Normally distributed measurement data are expressed as the means ± standard deviations (‾x ± s). Data without a normal distribution are presented as medians (P25, P75). Categorical variables are expressed as values and percentages (%). When appropriate, the independent-sample t test or Mann‒Whitney U test was used to compare continuous variables, while the chi‒square test was used to compare categorical variables. To screen clinical data, qualitative imaging characteristics, and quantitative parameters with statistical significance in predicting high Ki-67 expression in stage IA LUAD, the independent risk factors were determined using a stepwise method, a logistic regression model was established, and a nomogram was drawn. Internal and external validation groups were used to validate the model, and calibration and receiver operating characteristic (ROC) curves were used to assess the predictive capability and discriminative performance of the nomogram model. The goodness-of-fit of the model was determined using the Hosmer–Lemeshow test. Decision curve analysis was performed to determine the clinical net benefit threshold. Survival analysis was performed using R software with data from the training cohort (*n* = 193), and Kaplan–Meier survival curves were generated. Statistical significance was set at *P* < 0.05.

## Results

### Interobserver agreement

The interobserver Cohen’s kappa values ranged from 0.821 to 0.947, and the ICC was 0.852. The results showed favorable interobserver reproducibility for feature extraction.

### Patient characteristics and CT morphological characteristics

In the training group, a significantly greater proportion of patients with high Ki-67 expression were male, were older, had lesions with solid components, had clearer tumor–lung interfaces, had signs of lobulation and spiculation, had pleural indentation, and had a significantly larger maximum nodule diameter than patients with low Ki-67 expression (*P* < 0.05).

In the internal validation group, sex, lesion nature, tumor-lung interface, lobulation, and maximum nodule diameter differed significantly between patients with high and low Ki-67 expression (*P* < 0.05).

**Fig. 2:**
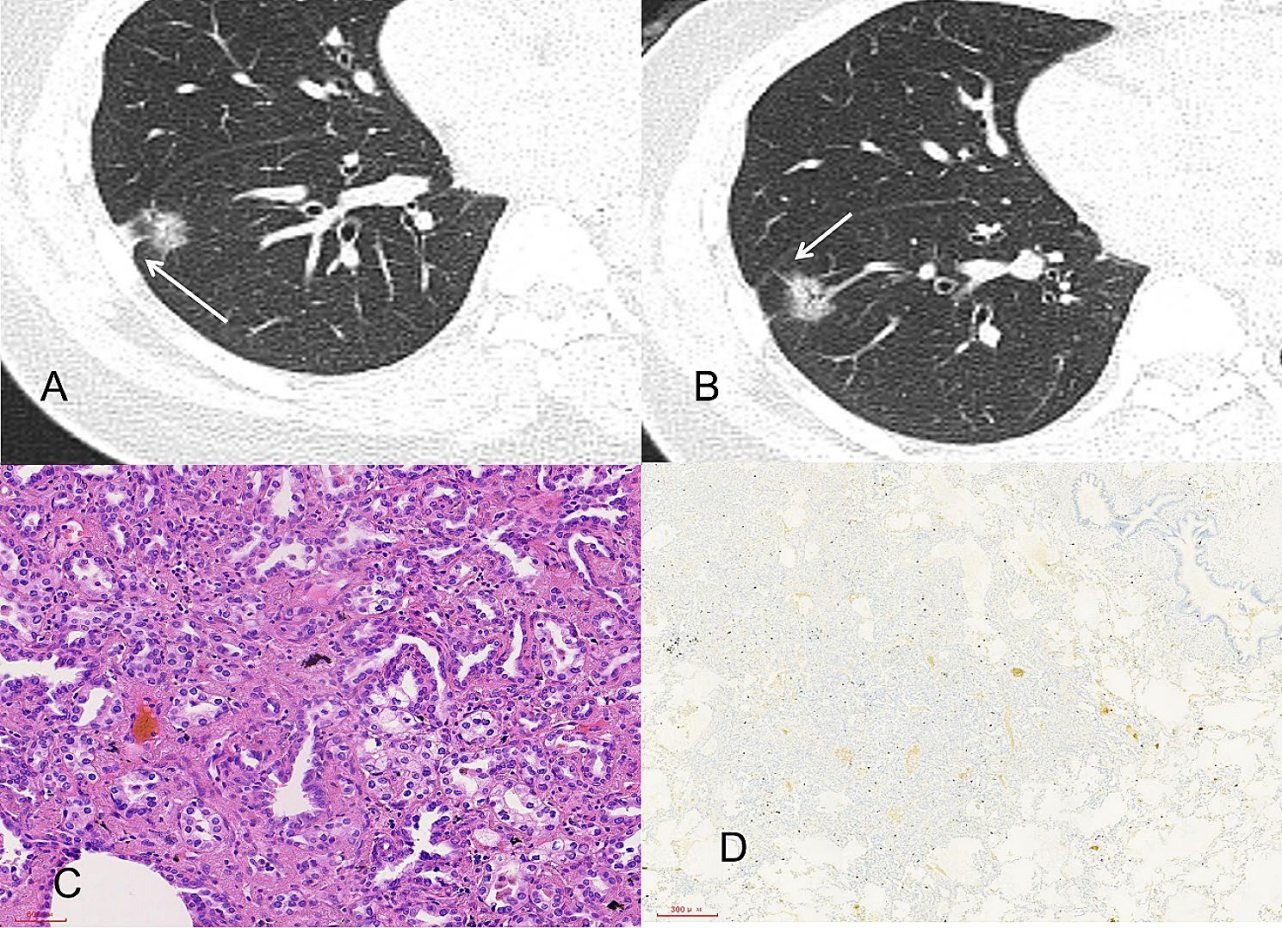
A middle-aged woman with stage IA lung adenocarcinoma. (**A, B**) Computed tomography images showing mixed ground-glass opacities in the right lower lobe, with clear boundaries; no lobulation, spiculation, or pleural indentation sign (→); and a bronchial air sign within the lesion, with a maximum nodule diameter of 2.54 cm. Pathological image showing invasive adenocarcinoma, with predominant acinar growth (hematoxylin and eosin staining ×200). (**D**) Pathological image showing low Ki-67 expression (Ki-67 immunohistochemistry ×40). According to the nomogram, the total score was 80, and the preoperative probability of high Ki-67 expression was approximately 9%. The postoperative pathological diagnosis was a Ki-67 index of 5%, indicating low Ki-67 expression

**Fig. 3:**
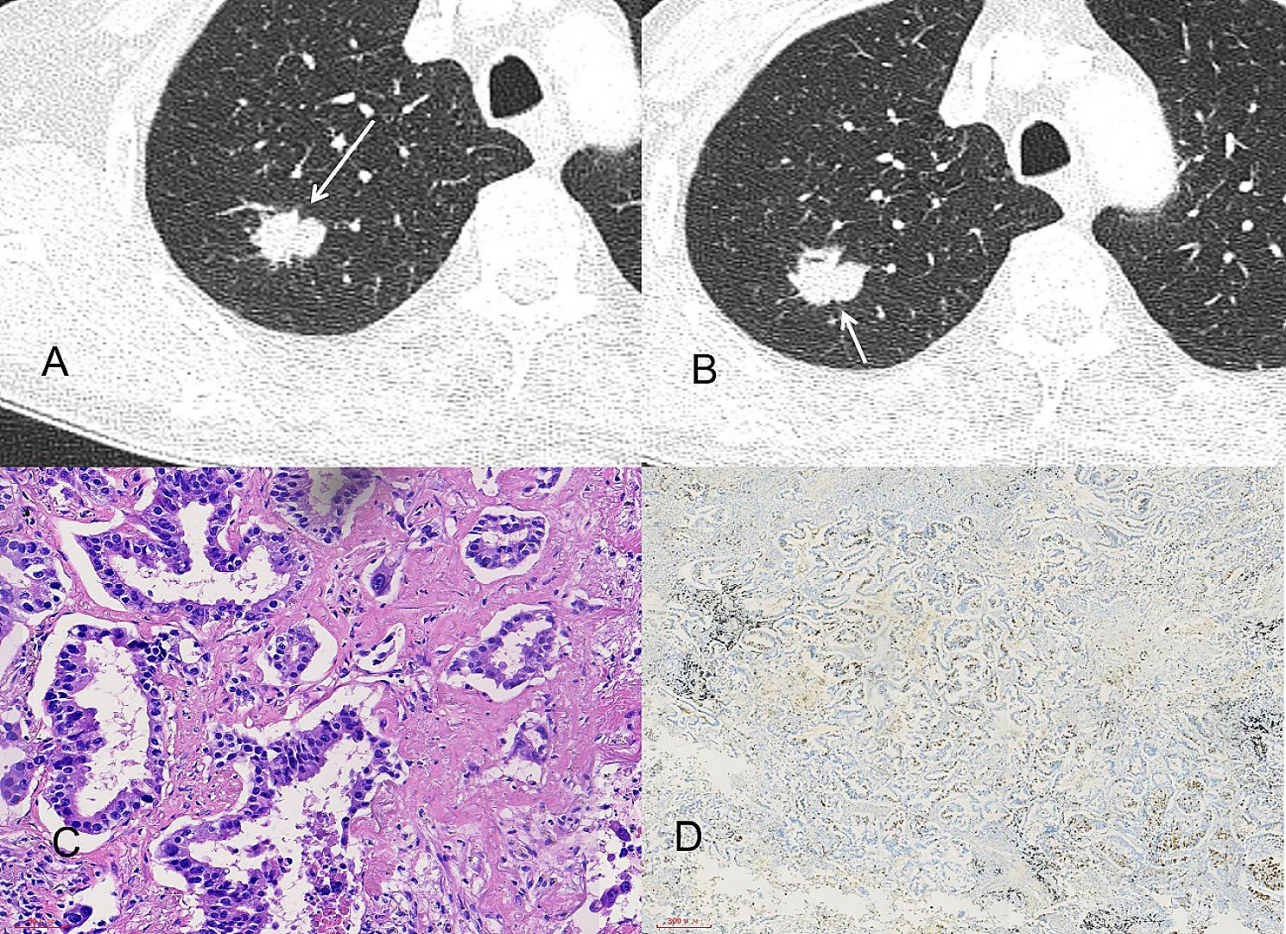
An elderly man with stage IA lung adenocarcinoma. (**A, B**) Computed tomography images showing a solid nodule in the right upper lobe, with shallow lobulation (→), spiculation, no pleural indentation sign, and a maximum nodule diameter of 2.12 cm. Pathological images showing invasive adenocarcinoma, predominantly of the solid type and partly of the acinar type (hematoxylin and eosin staining ×200). (**D**) Pathological image showing high Ki-67 expression (Ki-67 immunohistochemistry ×40). According to the nomogram, the total score was 224, and the probability of high Ki-67 expression was approximately 85%. The postoperative pathological diagnosis was a Ki-67 index of 25%, indicating high Ki-67 expression

In the external validation group, age, lesion nature, tumor-lung interface, lobulation, spiculation, pleural indentation, and maximum nodule diameter differed significantly between patients with high and low Ki-67 expression (Tables 1 and 2; Figs. 2 and 3).

**Table 1:** Comparisons of clinical characteristics between the low and high Ki-67 labeling index (LI) groups

Clinical characteristics	Training cohort				Internal validation cohort				External validation cohort			
	Low-Ki-67 LI group(*n* = 102)	High-Ki-67 LI group(*n* = 91)	χ^2^/Z	*P*	Low-Ki-67 LI group(*n* = 73)	High-Ki-67 LI group(*n* = 56)	χ^2^/Z	*P*	Low-Ki-67 LI group(*n* = 53)	High-Ki-67 LI group(*n* = 20)	χ^2^/Z	*P*
Sex			*χ*^*2*^ *=* 16.6	< 0.001			*χ*^*2*^ *=* 13.9	0.0002			*χ*^*2*^ *=* 3.0	0.081
Male	34	57			28	40			15	10		
Female	68	34			45	16			38	10		
Age[year, *M*(*Q*_1_,*Q*_3_)]	60.0(54.0, 67.3)	64.0(54.0, 71.0)	*Z*=-2.0	0.042	61.0(55.0, 67.5)	63.5(54.0, 68.8)	*Z*=-0.8	0.404	57.0(51.0, 65.0)	65.0(55.3, 68.0)	*Z*=-2.1	0.032
Symptoms			*χ*^*2*^ *=* 1.8	0.412			*χ*^*2*^ *=* 0.7	0.696				0.727^a^
Cough, Phlegm	36	33			28	18			15	7		
Chest tightness	32	27			18	17			2	1		
No	43	31			27	21			36	12		
Smoking history			*χ*^*2*^ *=* 0.01	0.922			*χ*^*2*^ *=* 3.0	0.085				0.326^a^
Yes	43	39			45	26			45	15		
No	59	52			28	30			8	5		

^a^ Fisher’s exact tests were used for categorical variables

**Table 2:** Computed tomography features of the low and high Ki-67 labeling index (LI) groups

CT feature	Training cohort				Internal validation cohort				External validation cohort			
	Low-Ki-67 LI group(*n* = 102)	High-Ki-67 LI group(*n* = 91)	χ^2^/Z	*P*	Low-Ki-67 LI group(*n* = 73)	High-Ki-67 LI group(*n* = 56)	χ^2^/Z	*P*	Low-Ki-67 LI group(*n* = 53)	High-Ki-67 LI group(*n* = 20)	χ^2^/Z	*P*
Lobe location			*χ*^*2*^ *=* 6.2	0.185				0.44^a^				0.285^a^
Left upper lobe	22	18			18	9			16	4		
Left lower lobe	24	11			15	7			8	2		
Right upper lobe	30	38			26	25			18	7		
Right middle lobe	9	6			4	4			0	2		
Right lower lobe	17	18			10	11			11	4		
Tumor density type			*χ*^*2*^ *=* 48.2	< 0.001			*χ*^*2*^ *=* 20.2	< 0.001				< 0.001^a^
Solid	39	78			26	40			3	13		
Mix ground-glass opacities	38	12			17	11			11	5		
Pure GGO	25	1			30	5			39	2		
Shape			*χ*^*2*^ *=* 0.8	0.659			*χ2 = 0.4*	0.857				0.160^a^
Round	36	28			24	19			25	5		
Oval	34	29			27	18			19	12		
Irregular	32	34			22	19			9	3		
Tumor lung interface			*χ*^*2*^ *=* 21.6	< 0.001			*χ*^*2*^ *=* 8.3	0.04				< 0.001^a^
Distinct	40	64			40	28			47	18		
Indistinct	62	37			33	18			9	2		
Lobulation			*χ*^*2*^ *=* 21.4	< 0.001			*χ*^*2*^ *=* 11.2	0.001				0.004^a^
Yes	29	56			24	35			21	16		
No	73	38			49	21			31	4		
Spiculation			*χ*^*2*^ *=* 9.2	0.002			*χ*^*2*^ *=* 3.8	0.071			*χ*^*2*^ *=* 4.1	0.044
Yes	45	60			37	38			18	12		
No	57	31			36	18			35	8		
Vacuole			*χ*^*2*^ *=* 1.7	0.197			*χ*^*2*^ *=* 1.5	0.215			*χ*^*2*^ *=* 1.8	0.186
Yes	44	31			38	20			25	6		
No	58	60			39	36			28	14		
Pleural indentation			*χ*^*2*^ *=* 7.3	0.007			*χ*^*2*^ *=* 2.2	0.194			*χ*^*2*^ *=* 4.5	0.033
Yes	56	67			43	40			25	15		
No	46	24			30	16			28	5		
Broncho vascular bundle sign			*χ*^*2*^ *=* 0.4	0.508			*χ2 = 0.001*	0.978			*χ*^*2*^ *=* 1.5	0.223
Yes	79	74			51	39			26	13		
No	23	17			22	17			27	7		
Air bronchogram			*χ*^*2*^ *=* 0.4	0.503			*χ*^*2*^ *=* 0.6	0.562			*χ*^*2*^ *=* 0.4	0.538
Yes	29	22			24	15			20	6		
No	73	69			49	41			33	14		
Maximum nodule diameter[cm, *M*(*Q*_1_,*Q*_3_)]	1.3(1.0, 1.8)	1.9(1.6, 2.3)	*Z*=-5.7	< 0.001	1.2(0.9, 1.6)	2.0(1.7, 2.4)	*Z*=-5.5	< 0.001	0.8(0.5, 1.2)	1.5(0.7, 2.0)	*Z*=-3.2	0.002

^a^ Fisher’s exact tests were used for categorical variables

### Establishment of the binary logistic regression model and nomogram

The best predictive model was obtained using forward stepwise logistic regression. The results showed that the independent preoperative predictive factors for high Ki-67 expression in stage IA LUAD included sex (odds ratio [OR] 0.342; 95% confidence interval [CI] 0.165–0.710), lobulation (OR 0.443; 95% CI 0.213–0.992), tumor density (OR 0.205; 95% CI 0.102–0.414), and maximum nodule diameter (OR 3.303; 95% CI 1.659–6.576) (Table 3).

**Table 3:** Logistic regression analysis of significant clinical characteristics and CT features of patients with stage IA lung adenocarcinoma with high Ki-67 expression

Independent predictive factors	β	OR	Odds ratio (95% CI)	*P*
Sex	-1.072	0.342	0.165 ~ 0.710	0.004
Lobulation	-0.814	0.443	0.213 ~ 0.922	0.030
Tumor density type	-1.582	0.205	0.102 ~ 0.414	< 0.001
Maximum nodule diameter	1.195	3.303	1.659 ~ 6.576	0.001

CI confidence interval

**Fig. 4:**
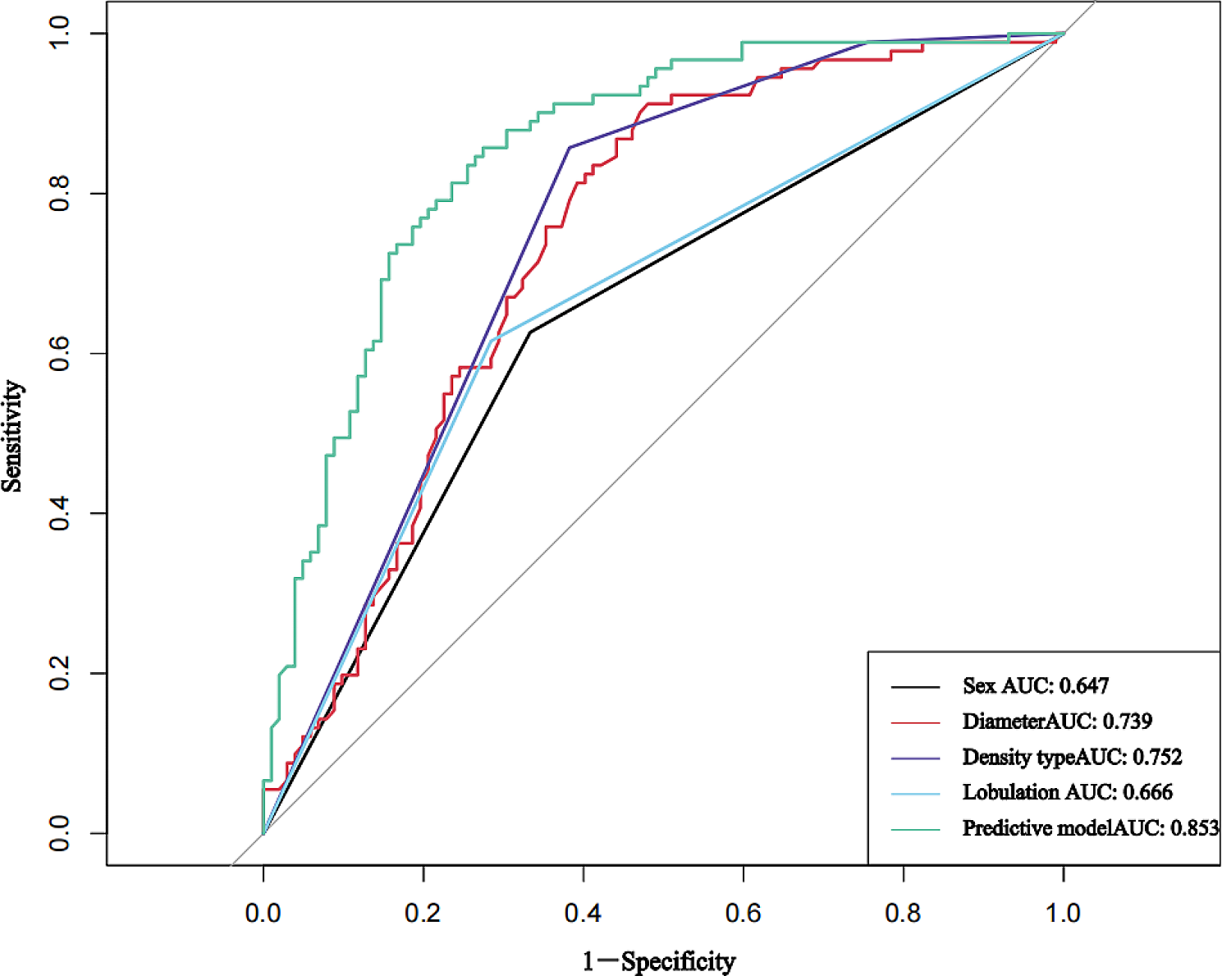
ROC curves of patient sex, tumor density type, lobulation, maximum nodule diameter and high Ki-67 expression in stage IA lung adenocarcinoma in the training cohort according to the predictive model. AUC, area under the receiver operating characteristic curve

A predictive model for high Ki-67 expression in LUAD was constructed based on clinical and MSCT features, with an area under the ROC curve of 0.853 (95% CI 0.799–0.907), a sensitivity of 85.7%, and a specificity of 72.5% (*P* < 0.001). The ROC analysis results for each independent predictive factor showed areas under the curve of 0.647 for sex, 0.752 for tumor density type, 0.666 for lobulation, and 0.739 for maximum nodule diameter, indicating that the predictive model had greater diagnostic efficiency (*P* < 0.001) (Fig. 4; Table 4).

**Table 4:** Receiver operating characteristic curve analysis to predict high Ki-67 expression in stage IA lung adenocarcinoma

Factors	Odds ratio (95% CI)	Cutoff value	Sensitivity	Specificity	*P*
Sex	0.647(0.568 ~ 0.725)	Male	66.7%	62.6%	< 0.001
Maximum nodule diameter	0.739(0.669 ~ 0.810)	> 1.3 cm	91.2%	52.0%	< 0.001
Tumor density type	0.752(0.682 ~ 0.821)	mGGO	61.8%	85.7%	< 0.001
Lobulation	0.666(0.588 ~ 0.743)	Yes	71.6%	61.5%	< 0.001
Predictive model	0.853(0.799 ~ 0.907)	> 0.4	85.7%	72.5%	< 0.001

CI, confidence interval

**Fig. 5:**
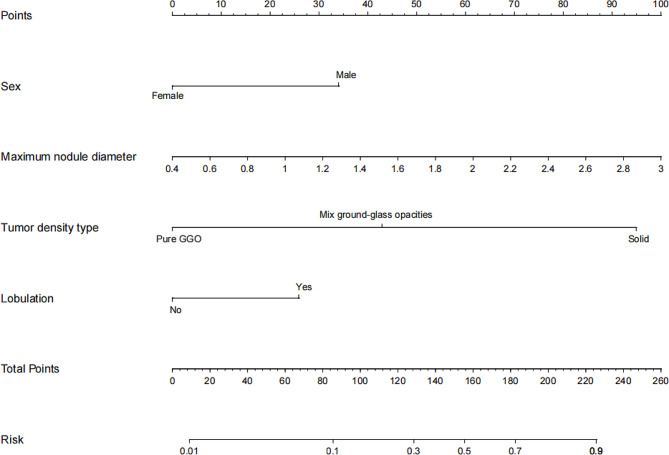
Nomogram model based on clinical and computed tomography features for predicting high Ki-67 expression in stage IA lung adenocarcinoma. According to the four indexes (sex, tumor density type, tumor lobulation, and maximum nodule diameter) of each patient, the vertical lines between each index and the nomogram points were drawn to obtain the score for each index. The scores of the five indicators are then summed to obtain the total score. Finally, a vertical line is drawn between the total score and the nomogram risk to predict the probability of high Ki-67 expression in stage IA lung adenocarcinoma

**Fig. 6:**
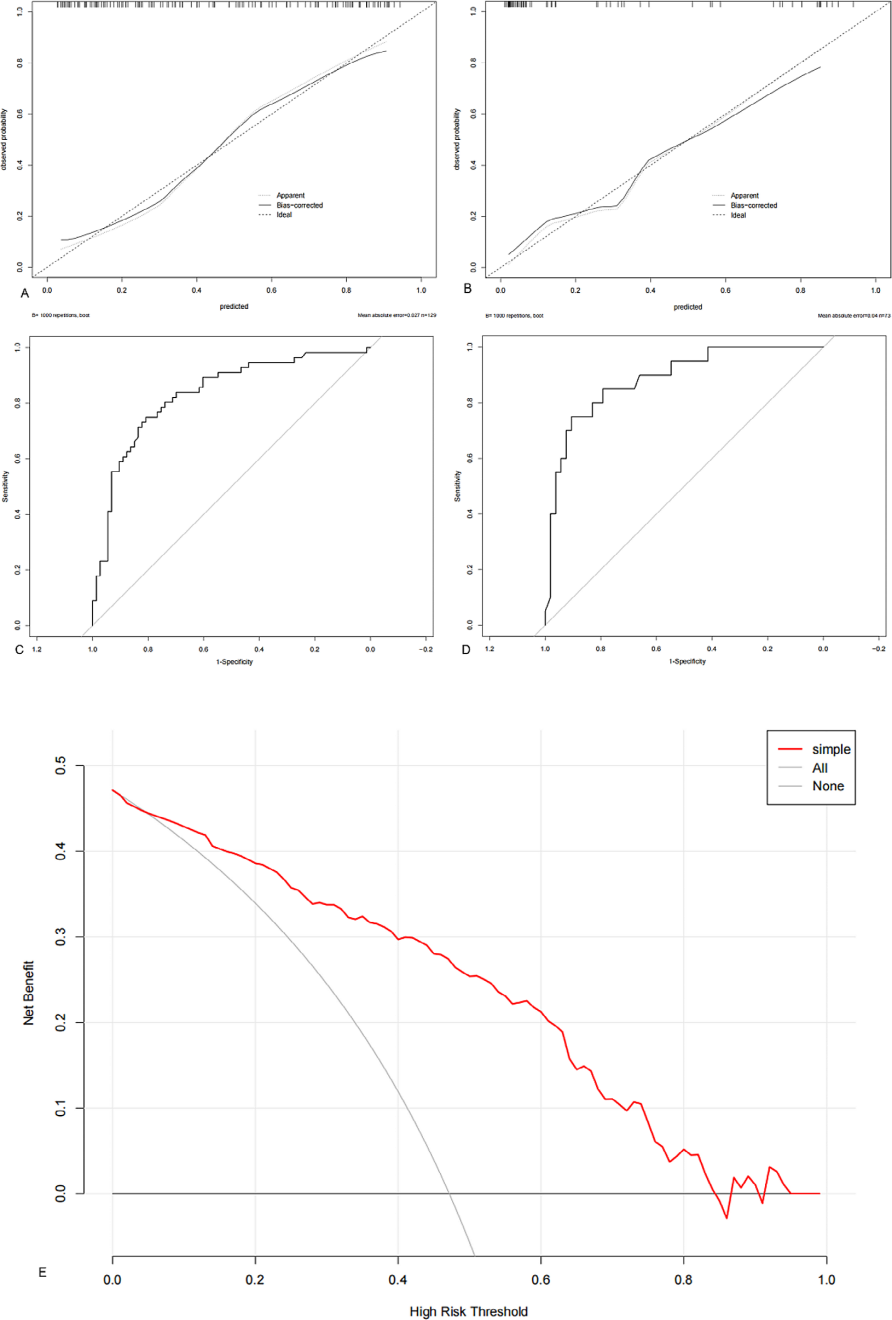
Calibration curve of the nomogram model based on clinical and CT features for predicting high Ki-67 expression in stage IA lung adenocarcinoma. Internal (**A**) and external (**B**) validation show a well-calibrated curve between the predictive model and actual observations, indicating the high discrimination and accuracy of the model. Receiver operating characteristic curves of the nomogram prediction model for internal (**C**) and external (**D**) validation. Decision curve analysis to evaluate the clinical net benefit of the preoperative prediction model for high Ki-67 expression in stage IA lung adenocarcinoma. Decision curve analysis (E) was used to evaluate the clinical net benefit of the preoperative prediction model for high Ki-67 expression in stage IA lung adenocarcinoma

A nomogram for predicting Ki-67 expression in stage IA LUAD was successfully established using R software (Fig. 5). After internal validation, a high-quality calibration curve was obtained (Fig. 6A), indicating good consistency between the predictive model and the actual observations. The area under the ROC curve was 0.853, indicating good discrimination. The results of the Hosmer–Lemeshow test indicated a good fit for the model (*P* = 0.653).

The results of the external validation showed a well-calibrated curve between the predictive model and the actual observations (Fig. 6B). The areas under the ROC curve for internal and external validation were 0.833 and 0.886, respectively (Fig. 6C and D), indicating the high discrimination and accuracy of the model.

The decision curve for the nomogram is shown in Fig. 6E. The net benefit of the decision curve for the predictive nomogram was greater than that for assuming that all patients had high Ki-67 expression when the threshold probability was in the range of 0.04–0.84. This suggests that a therapeutic strategy based on our nomogram will improve clinical outcomes.

### Ki-67 LI and RFS

**Fig. 7:**
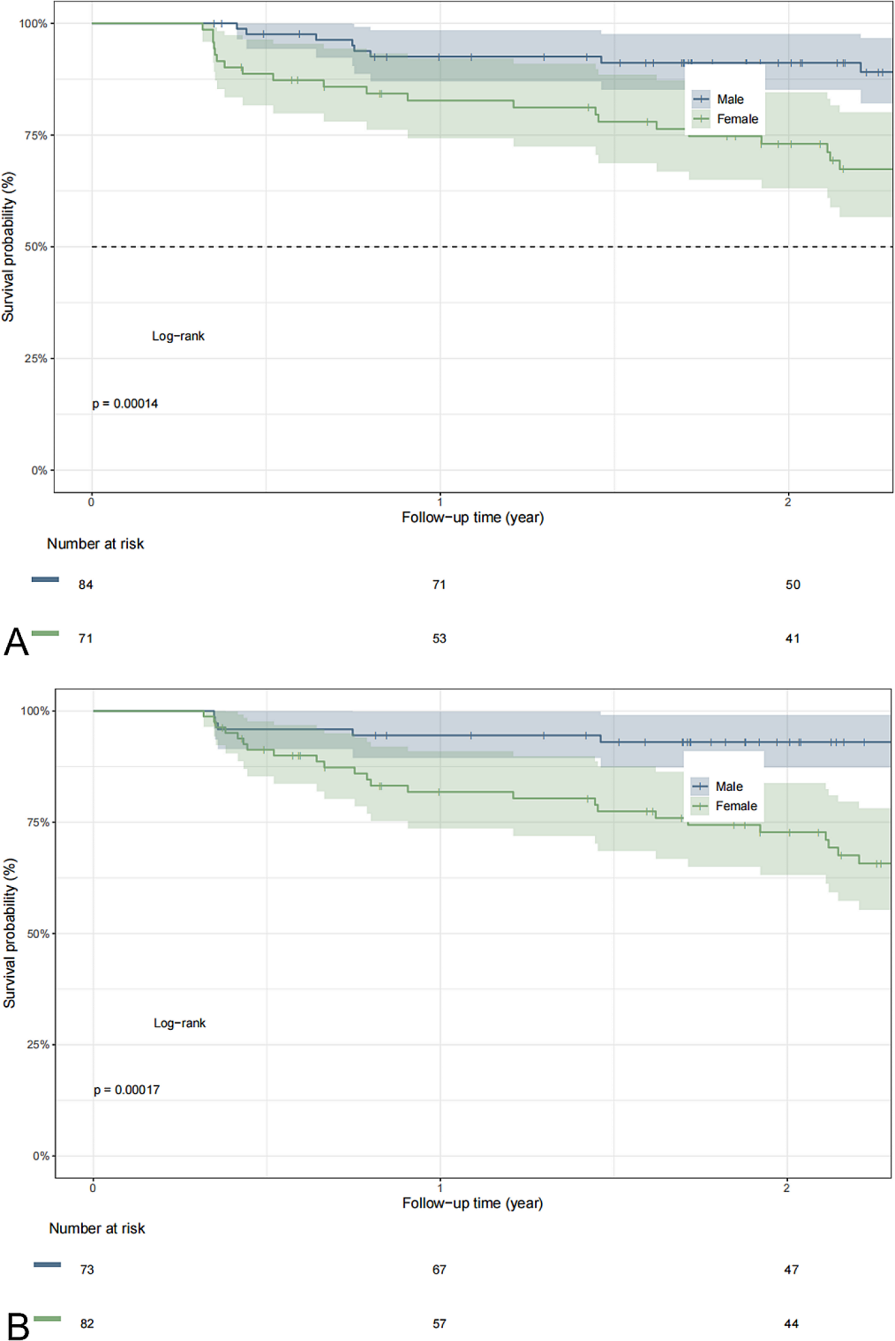
Kaplan–Meier curves depicting recurrence-free survival according to the pathological Ki-67 labeling index (LI) (**A**) and the predicted Ki-67 LI (**B**). After stratifying by the Ki-67 LI (low or high), patients with both pathological and predicted high Ki-67 LI values had poorer RFS than patients with low Ki-67 LI values (*P* < 0.001)

The RFS curves scaled by pathological and predicted Ki-67 LI based on our nomogram using Kaplan–Meier analysis are shown in Fig. 7A. Pathologically, patients with a high Ki-67 LI had poorer RFS than patients with a low Ki-67 LI (*P* < 0.001). The 1- and 2-year RFS rates were 82.7% and 73%, respectively, in the high-Ki-67 LI group and 92.6% and 91.2%, respectively, in the low-Ki-67 LI group. Similar results were obtained using the nomogram. The application of the nomogram is shown in Fig. 7B.

## Discussion

Currently, the Ki-67 index is commonly regarded as a useful biomarker in clinical practice for predicting tumor cell proliferation, invasiveness, and prognosis and provides significant value in early lung cancer diagnosis and treatment assessment [21, 22]. Recent research findings indicate that a Ki-67 index of 0.10–0.15 is a pivotal threshold for the proliferative activity of lung adenocarcinoma [20], suggesting a turning point toward higher tumor grades. Therefore, in this study, the Ki-67 threshold was set to 0.15 to categorize high and low expression levels.

### Analysis of clinical data and MSCT features of Ki-67 expression in stage IA lung adenocarcinoma

In this study, individuals with high Ki-67 expression in stage IA lung adenocarcinoma were significantly more often male and older and had a larger maximum nodule diameter (> 1.33 cm). Ma et al. [23] evaluated CT features and Ki-67 expression in stage IA lung adenocarcinoma using a threshold of 0.1 and reported strong correlations between patient sex, tumor length and width, and Ki-67 expression. They also observed significantly higher Ki-67 expression in male patients with stage IA lung adenocarcinoma [24], consistent with the results of the present study.

Individuals with high Ki-67 expression in stage IA lung adenocarcinoma in this study were more likely to exhibit mGGOs or solid nodules. According to the natural growth pattern of lung adenocarcinoma, early-stage tumors are typically characterized by ground-glass-density nodules that gradually increase in size, followed by the emergence of solid components within the lesions. A greater proportion of solid components within ground-glass opacities is associated with greater invasiveness and poorer prognosis [25]. Lesions with clear tumor-lung interfaces, as well as the presence of lobulation, spiculation, and pleural indentation signs, are more likely to exhibit high Ki-67 expression. Zhang et al. [26] demonstrated that a clear tumor-lung interface is a risk factor for invasive pGGO lung adenocarcinoma. Tumors with high Ki-67 expression often exhibit irregular morphology owing to inconsistent cell growth rates and differentiation levels at different locations along the tumor periphery. This irregular growth may result in lobulated shapes, and differences in fibrous tissue contraction within the tumor may increase the likelihood of spiculation and pleural indentation. Ma et al. [23] reported correlations between lobulation, spiculation, lesion nature, air bronchogram signs, vessel convergence signs, pleural indentation signs, lung tissue fibrosis, and Ki-67 expression. Huang et al. [27] reported associations between Ki-67 expression and tumor diameter, tumor density, spiculation, lobulation, and bronchial inflation. Yanagawa et al. [28] discovered that malignant ground-glass opacities often exhibit lobulated growth and that the presence of lobulation signifies poor patient prognosis.

### Analysis of the logistic regression results and predictive performance and application of the prediction model

Research on preoperative models for predicting Ki-67 expression in stage IA lung adenocarcinoma is limited. Liu et al. [15] analyzed the clinical and CT features of 376 patients with early-stage lung adenocarcinoma and reported that male sex, carcinoembryonic antigen positivity, spiculation, vessel convergence, and the proportion of solid tumor components were associated with high Ki-67 expression. Fu et al. [29] used radiomics to construct a nomogram and confirmed that smoking history and neuron-specific enolase combined with radiomic scores could predict Ki-67 expression. The present study identified male sex (OR 0.343; 95% CI 0.165–0.71), lobulation (OR 0.443; 95% CI 0.213–0.992), tumor density (OR 0.205; 95% CI 0.102–0.414), and maximum nodule diameter (OR 3.303; 95% CI 1.659–6.576) as independent predictive indicators of high Ki-67 expression in stage IA lung adenocarcinoma. The established nomogram, which was validated internally and externally, further demonstrated high accuracy, making it more universally applicable and practical than the methods proposed in the aforementioned studies. The nomogram model in the present study visualizes the regression equation in a graphical format, providing a convenient and intuitive tool for clinicians to calculate the probability of high Ki-67 expression in stage IA lung adenocarcinoma and to facilitate personalized predictions. The ROC curves for the training set and the internal and external validation sets showed that the model had high discriminative ability and accuracy. The results of the calibration and decision curve analyses suggested the good consistency and clinical utility of the model.

The results of a recent phase III clinical study revealed a greater local recurrence rate after segmentectomy (10.5% vs. 5.4%) than after lobectomy in early-stage NSCLC (tumor diameter ≤ 2 cm, CT tumor solid component ratio > 0.5). However, the 5-year overall survival rate was greater after segmentectomy than after lobectomy (94.3% vs. 91.1%) [30]. Although the local recurrence rate was greater after segmentectomy in this study, segmentectomy preserved more lung tissue, which is beneficial for potential postoperative treatments for recurrence, other cancers, or life-threatening diseases, resulting in a relatively greater overall survival rate after segmentectomy. Therefore, the results of the present study suggested that segmentectomy should be the preferred surgical procedure for early-stage NSCLC. Some studies have reported that the expression level of Ki-67 and tumor stage affect PFS, which is consistent with the results of this study [10].

This study utilized the comprehensive features of CT images of lesions to develop a predictive model for Ki-67 expression in stage IA lung adenocarcinoma to determine its clinical usefulness. Decision curve analysis was applied in this study to confirm the predictive value of the predictive model, and both internal and external validation were performed in this study to provide more insight into the generalizability of the combined model. It is widely acknowledged that the heterogeneity of tumor biology leads to varied prognoses reflecting the metastatic or invasive behavior of a particular tumor [8]. Therefore, this study aimed to avoid the impact of tumor heterogeneity on the accuracy of the predictive model. We assessed the predictive accuracy of Ki-67 expression levels, as determined by our model, in estimating the prognosis of stage IA lung adenocarcinoma. Patients predicted to have a high Ki-67 LI by our nomogram exhibited worse RFS than those with a low Ki-67 LI. Compared to preoperative biopsies and rapid intraoperative Ki-67 immunohistochemistry, this approach can predict tumor proliferative activity and identify the risk of recurrence preoperatively, aiding clinicians in selecting appropriate surgical approaches. This model has the potential to improve postoperative survival rates and reduce recurrence rates in patients with early-stage lung cancer. To further validate the clinical utility of this model, future research will explore its application in the choice of surgical approach and its effects on postoperative survival and recurrence rates and whether the model can be used to evaluate the choice of adjuvant targeted therapy options. Due to the lack of biological interpretability, the application of radiomic models in clinical settings is hindered [31]. Compared with radiomics, the interpretation of lesion features in CT scans has more advantages in clinical applications. Naturally, we will also include radiomics in our future research to achieve greater accuracy.

This study has two limitations. First, due to the relatively limited sample size, it was impossible to completely avoid confounding and selection biases. Second, this study was retrospective, and differences in CT equipment and scanning parameters may have affected the results.

In conclusion, clinical and MSCT features, including sex, maximum nodule diameter, lesion nature, and lobulation, allow differentiation between high and low Ki-67 expression in stage IA lung adenocarcinoma. The combined diagnostic model and nomogram established in this study, along with the nomogram, provide improved preoperative predictions of Ki-67 expression, offering significant value in the diagnosis, treatment, and prognostic assessment of early-stage lung cancer.

## Data Availability

The datasets generated or analyzed during the study are available from the corresponding author on reasonable request.
